# MSBiLSTM-Attention: EEG Emotion Recognition Model Based on Spatiotemporal Feature Fusion

**DOI:** 10.3390/biomimetics10030178

**Published:** 2025-03-13

**Authors:** Yahong Ma, Zhentao Huang, Yuyao Yang, Zuowen Chen, Qi Dong, Shanwen Zhang, Yuan Li

**Affiliations:** 1 Xi’an Key Laboratory of High Precision Industrial Intelligent Vision Measurement Technology, School of Electronic Information, Xijing University, Xi’an 710123, China; yahongma@sina.com (Y.M.); yyy11171999@outlook.com (Y.Y.); 17696749100@163.com (Z.C.); wjdw716@163.com (S.Z.); 2 School of Mathematics and Statistics, Zhengzhou University, Zhengzhou 710003, China; qid004841@gmail.com; 3 School of Physics and Electronic-Electrical Engineering, ABA Teachers College, Wenchuan 623002, China

**Keywords:** emotion recognition, multi-scale, convolutional neural network (CNN), bidirectional long short-term memory (Bi-LSTM), attention mechanism

## Abstract

Emotional states play a crucial role in shaping decision-making and social interactions, with sentiment analysis becoming an essential technology in human–computer emotional engagement, garnering increasing interest in artificial intelligence research. In EEG-based emotion analysis, the main challenges are feature extraction and classifier design, making the extraction of spatiotemporal information from EEG signals vital for effective emotion classification. Current methods largely depend on machine learning with manual feature extraction, while deep learning offers the advantage of automatic feature extraction and classification. Nonetheless, many deep learning approaches still necessitate manual preprocessing, which hampers accuracy and convenience. This paper introduces a novel deep learning technique that integrates multi-scale convolution and bidirectional long short-term memory networks with an attention mechanism for automatic EEG feature extraction and classification. By using raw EEG data, the method applies multi-scale convolutional neural networks and bidirectional long short-term memory networks to extract and merge features, selects key features via an attention mechanism, and classifies emotional EEG signals through a fully connected layer. The proposed model was evaluated on the SEED dataset for emotion classification. Experimental results demonstrate that this method effectively classifies EEG-based emotions, achieving classification accuracies of 99.44% for the three-class task and 99.85% for the four-class task in single validation, with average 10-fold-cross-validation accuracies of 99.49% and 99.70%, respectively. These findings suggest that the MSBiLSTM-Attention model is a powerful approach for emotion recognition.

## 1. Introduction

Emotions significantly influence human cognition, decision-making, and daily actions [1,2]. They are not only closely related to an individual’s inner feelings but also interact with physiological responses. Emotion recognition technology is crucial for brain–machine interface research and is vital in areas like human–computer interaction, user emotion detection [3], gaming and entertainment [4], and healthcare. There are two main types of emotion detection methods. The first type relies on non-physiological cues, such as facial expressions [5], body posture [6], eye blinking [7], and voice characteristics [8]. However, these methods can be uncertain and unreliable since individuals may hide their true feelings. The second type depends on physiological indicators, including EEG, ECG, EMG, EOG, skin conductance, respiratory rate, and so on. Physiological factors usually play an important role in emotion recognition, but the expression and perception of emotions are also significantly influenced by external environment and cultural background.

As a physiological method, EEG is extensively utilized in emotion recognition tasks due to its ease of acquisition and high temporal resolution. The EEG signals are directly captured at the cortical surface. Since emotions are often strongly related to neuronal firing activities within the brain’s structures, EEG can effectively reflect emotional states and provide a finer granularity of emotional detail [9]. Furthermore, thanks to its high temporal precision and non-intrusive nature, EEG-based emotion recognition technology has been widely recognized and has achieved good results in practical applications [10]. Despite its significant advantages in revealing genuine human emotions, this technology still faces a number of difficulties, including the intricacy of signal processing, subject variability, and noise disturbance [11,12,13,14,15]. Therefore, the development of methods that can accurately and reliably interpret EEG-based emotion signals is crucial for enhancing the precision and dependability of an emotional recognition system.

Over the past few years, the rapid development of machine learning and deep learning technologies has propelled the progress of emotion recognition according to EEG signals, which has been applied across various fields. Machine learning mainly includes traditional supervised learning methods, such as discriminant analysis [16], support vector machine (SVM) [17], nearest neighbor [18], Bayesian method [19], and perceptron [20]. The general process of emotion detection consists of emotion triggering, signal acquisition, feature extraction, and classification recognition. However, most emotion recognition methods using machine learning depend significantly on handcrafted feature extraction, which significantly impacts the accuracy of recognition. Nevertheless, with the continuous development of artificial intelligence in recent years, deep learning, leading to its advantage of automatic feature extraction, has achieved end-to-end classification [21], and computational models that learn the mathematical representation of input data through continuous nonlinear transformations [22] are increasingly used in emotion recognition. Li et al. [23] designed a spatial-temporal-connective multi-scale convolutional neural network (STC-CNN) specifically to recognize emotion from EEG signals; the average accuracy is up to 96.79% and 96.89% in categorizing the dimensions of valence and arousal, respectively. Li et al. [24] presented a graph-regularized sparse linear regression (GRSLR) to overcome the issues in EEG emotion recognition. The main idea of GRSLR is to use the transformation matrix in linear regression. Tao et al. [25] introduced an attention-based convolutional recurrent neural network (ACRNN) to improve the ability of feature extraction for better discrimination from EEG signals and to enhance the accuracy of emotion recognition. Lu et al. [26] proposed a two-dimensional convolutional multilayer perceptron network (CMLP-Net) based on EEG signals. CMLP-Net includes time-stream shared convolution, time-thinning spatio-temporal convolution, and spatial interactive multi-layer perceptron (MLP). Fan et al. [27] proposed a two-module EEG emotion recognition method based on an improved capsule network and a residual long short-term memory network (ResLSTM). Firstly, the enhanced capsule network, serving as a spatial module, facilitates the learning of specific EEG spatial representations. Subsequently, the ResLSTM in the temporal module inherits the information flow from the preceding spatial module. Through residual connections, complementary learning of the features from both spatiotemporal modules is conducted, thereby obtaining more discriminative EEG features and ultimately enhancing the model’s classification capabilities. Despite the immense potential demonstrated by deep learning in EEG signal processing, there remain unresolved challenges and issues that await further attention. Traditional convolution operations focus on local spatial correlation and utilize fixed-size filters, but in modern CNNs, the parameters of these filters are automatically learned through the training process, eliminating the need for manual setting. Traditional recurrent neural networks (RNNs) have certain advantages in processing time series data, but their inherent multi-layer recurrent structure may lead to issues such as gradient explosion or vanishing, which limits their ability to effectively capture and process long-sequence information. To effectively alleviate the issues present in RNNs, long short-term memory (LSTM) networks introduce gating mechanisms. However, LSTM only considers unidirectional dependencies in sequential data, meaning that information can only flow forward or backward, but not both. Although existing research on emotion recognition based on EEG signals has achieved relatively high classification accuracy, the accuracy varies across different application scenarios and is highly dependent on the features of the datasets used. Additionally, there is a lack of consistency in the standards for selecting electrode channels among studies, which is also a significant factor affecting classification accuracy and broader applicability. Standard convolution is simple in structure, easy to implement and optimize, and it may lead to feature loss or inaccuracy when dealing with highly variable and complex data, thereby affecting the final task performance. Therefore, this study proposes a model that combines multi-scale convolution, bidirectional long short-term memory (Bi-LSTM) networks, and attention mechanisms for emotion recognition and classification grounded in electroencephalogram signals. In comparison with the prior studies, the novelty of this study lies in the following:

(1) Unprocessed EEG signals are directly fed into, which simplifies the operation and reduces the complexity of data processing;

(2) Multi-scale convolution can understand input data from different perspectives and scales, enhancing the model’s generalization ability;

(3) The Bi-LSTM structure outperforms traditional LSTM models by effectively solving the issues of gradient explosion and vanishing, improving the ability to learn from time series data, especially in capturing sequential context information;

(4) The attention mechanism enables the model to automatically identify and concentrate on important electrode channel features, avoiding the cumbersome and subjective process of manual feature selection and improving the effectiveness of feature extraction;

(5) The model has been extensively tested on the public datasets, which are SEED and SEED-IV emotion recognition datasets, demonstrating excellent performance and validating its effectiveness and robustness in practical applications.

## 2. Materials and Methods

### 2.1. SEED Dataset

**Figure 1:**
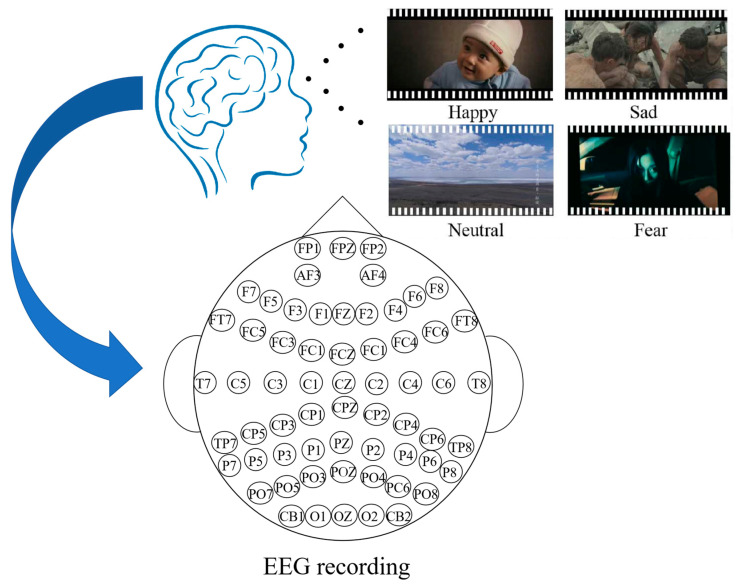
Experimental dataset collection process.

The emotion EEG dataset (SEED) is supplied by the BCMI Laboratory of the Shanghai Jiao Tong University. SEED includes EEG recordings from 15 participants [28,29]. Within the experimental period, 15 Chinese movie clips were selected as stimuli, encompassing positive, neutral, and negative emotions. Each film lasts approximately 4 min. Each segment has been meticulously refined to elicit distinct emotional responses and amplify the affective impact. Each participant undergoes 15 trials in each experiment. Each clip is preceded by a 5 s prompt, 45 s for self-assessment, followed by a 15 s break. To obtain feedback, participants were asked to immediately complete a questionnaire after watching each film, reporting their emotional reactions to the clips. SEED-IV [30] consists of EEG recordings from 15 subjects and includes 72 carefully selected movie clips, which aim to provoke emotional responses of happiness, sadness, fear, or neutrality, similar to SEED. The data collection process is shown in Figure 1. To test the stability and portability of emotion recognition models, we tested SEED and SEED-IV separately. Because of the large volume of data, 1000 continuous recordings were extracted from each participant’s video segment in our experiment. Therefore, SEED and SEED-IV extracted 15 participants × 15 videos × 1000 EEG data sets = 225,000, and 15 participants × 24 videos × 1000 EEG data sets = 360,000, respectively. Thus, we obtained a total of 225,000 EEG data sets from SEED and 360,000 from SEED-IV. The data and category labels in the dataset are uniformly distributed and balanced.

### 2.2. CNN-Bi-LSTM-Attention Model

An obvious characteristic of electroencephalogram (EEG) signals is their strong correlation over time series. Bi-LSTM has proven to be a suitable choice as it is good at capturing and analyzing long-term dependencies of time series, effectively extending features and processing sequential data along the temporal dimension. While Bi-LSTM performs exceptionally well in time series analysis, its limitations in extracting spatial features cannot be overlooked. Multi-scale convolution, on the other hand, can capture various characteristics of spatial and frequency bands in EEG signals through filters of different scales. Small-scale filters concentrate on the precise extraction of local features, while large-scale filters excel at capturing broader contextual information. This design can enhance the ability to learn spatial features and improve its sensitivity to the underlying nonlinear relationships and multifrequency dynamics of EEG signals.

**Figure 2:**
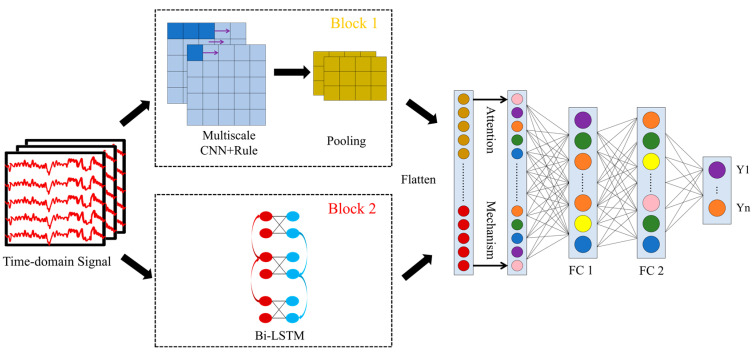
The architecture of the MSBiLSTM-Attention model.

The proposed model in this study not only integrates multi-scale convolution with Bi-LSTM but also adds an attention mechanism, forming a comprehensive MSBiLSTM-Attention model. This design draws on successful experiences from computer vision and machine translation, where the attention mechanism, as a powerful tool, dynamically highlights the most crucial spatiotemporal features and electrode channel information, thereby precisely guiding the model’s focus more important features. The proposed MSBiLSTM-Attention model includes an input layer, a multi-scale convolution layer, a Bi-LSTM layer, an attention mechanism layer, two fully connected layers, and an output layer. The multi-scale convolution layer and the Bi-LSTM layer correspond to two parallel modules, block1 and block2, respectively. The input layer first normalizes the raw data into time domain signals. Given that the EEG signal is one-dimensional, a one-dimensional CNN is employed to extract spatial features, whereas Bi-LSTM is utilized to capture temporal features. These features are subsequently concatenated and fed into an attention mechanism layer. Through computation, the attention mechanism assigns a weight to each feature channel. These weighted features are then further refined and dimensionally reduced by two fully connected layers. Ultimately, the Softmax function is applied to classify the results. The architecture of the MSBiLSTM-Attention model is shown in Figure 2.

#### 2.2.1. Convolutional Neural Network (CNN)

The CNN is a kind of feedforward neural network, in which artificial neurons are capable of responding to a subset of neighboring units. It performs superbly well in large-scale image processing. LeCun et al. [31] utilized CNNs for handwritten digit recognition back in the last century, but at that time, they were constrained by the limited computational capabilities of computers. However, with the ongoing enhancement of computational power, the training of deep neural networks has become increasingly easier, and the advantages of CNNs have progressively become apparent. The network structure of a CNN as described elsewhere, includes convolutional layers, pooling layers, and fully connected layers. The convolutional layer is notable for its weight sharing and sparse connectivity, primarily responsible for extracting local features from the input data. The pooling layer is usually placed after the convolutional layer, serving to decrease the dimensionality of the feature maps and decrease the computational load while preserving important feature information. The fully connected layer consolidates the features extracted by the convolutional and pooling layers, applies linear transformations through weights and biases, and then passes them through an activation function to perform nonlinear transformations, ultimately obtaining classification results or regression values. To identify the nonlinear features of the EEG signal, nonlinear activation functions (such as ReLU) are typically utilized for the convolutional layers in this paper. These functions have the benefits of avoiding gradient vanishing and lowering overfitting. The function of ReLU is represented by Equation (1):

(1) y=max(0,x)

Although the ReLU function solves the gradient-vanishing problem, the gradient is zero for negative values, which may result in some neurons remaining untrained, thereby reducing the expressive power of the network. To solve this issue, Maas et al. [32] introduced Leaky ReLU, which employs a small slope rather than the zero slope of ReLU for negative inputs, making the activation function smoother and more expressive. Consequently, it has been widely used in practical applications. The definition of Leaky ReLU is given by Equation (2):

(2) $$y={\{}_{ax,\;x<0}^{x,\;x\geq 0}$$

#### 2.2.2. Multi-Scale Networks

Generally speaking, simple feature extraction modules are often composed of convolutional neural networks, long short-term memory networks, and other basic blocks stacked together. These modules extract features from the lower layers of the network, and then these features pass through each layer to the top layer to obtain the final features. It is commonly understood that the features extracted at each layer capture information across different scales. Low-level features are more similar in local details, while high-level features are more similar in terms of semantics [33].

In various pattern-recognition fields [34,35], combining features from different depths serves as an excellent supplement to deep features to address the issue of multi-scale feature fusion. During the convolution process, employing convolution kernels of various sizes helps avoid network redundancy. Convolutional network layers with smaller scales possess smaller receptive fields and capture lower-level information, while those with larger scales are capable of capturing higher-level semantic information, enabling more accurate large-scale detection. Therefore, utilizing multi-scale convolution kernels for feature extraction is a successful strategy to boost the performance of EEG signal classification [36,37,38,39].

#### 2.2.3. Bidirectional Long Short-Term Memory

As a type of recurrent neural network, LSTM networks are widely employed in sequence data processing tasks such as speech recognition, natural language processing, and time series prediction.

Unlike RNN, LSTM is intended to capture long-term dependencies within input sequences. For LSTM, the computation of each element in the input sequence is performed first, as shown in Equation (3).

(3) $$f_{t}=\sigma \left(W_{\mathrm{if}}x_{t}+b_{\mathrm{if}}+W_{\mathrm{hf}}h_{t-1}+b_{\mathrm{hf}}\right)$$

Here, *h_t_*_−1_ represents the hidden state of the previous sequence, and *x_t_* is the current input vector to the LSTM neuron. The output of the forget gate, *f_t_*, is obtained from the sigmoid activation function.

The input gate is composed of two components. The sigmoid activation function and tanh activation function are used respectively, outputting *i_t_* and *g_t_*, as shown in Equations (4) and (5):

(4) $$i_{t}=\sigma \left(W_{\mathrm{ii}}x_{t}+b_{\mathrm{ii}}+W_{\mathrm{if}}h_{t-1}+b_{\mathrm{hi}}\right)$$

(5) $$g_{t}=\tanh\left(W_{\mathrm{ig}}x_{t}+b_{\mathrm{ig}}+W_{\mathrm{ig}}h_{t-1}+b_{\mathrm{hg}}\right)$$

The cell state *c_t_* consists of two parts. The first part is the product of the previous cell state *c_t_*_−1_ and the output of the forget gate *f_t_*, while the second part is its product with *g_t_*.

(6) $$c_{t}=f_{t}⊙c_{t-1}+i_{t}⊙g_{t}$$

(7) $$o_{t}=\sigma \left(W_{\mathrm{io}}x_{t}+b_{\mathrm{io}}+W_{\mathrm{ho}}h_{t-1}+b_{\mathrm{ho}}\right)$$

(8) $$h_{t}=o_{t}⊙\tanh\left(c_{t}\right)$$

**Figure 3:**
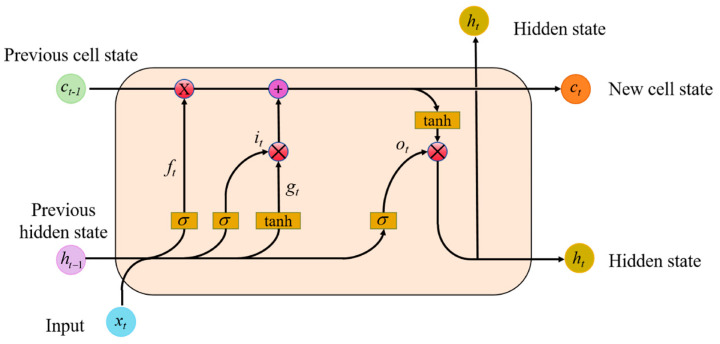
LSTM model.

As depicted in Figure 3, the update of the hidden state *h_t_* involves two components. The first component is *o_t_*, which is calculated from the previous sequence’s hidden state *h_t_*_−1_, the current sequence’s data *x_t_*, and the sigmoid activation function. The second part comprises the hidden state *c_t_* and the tanh activation function.

Although LSTM is proficient at handling long-term sequence information, it falls short in capturing local contextual information. EEG signals are electrical activity sequences recorded by different electrodes placed on the scalp, demonstrating significant temporal correlation. A bidirectional LSTM encodes the input time series. The output layer receives both past and future information from the input sequence through this structure. During training, the network weights are adjusted through forward and backward propagation.

#### 2.2.4. Fully Connected Layer (FC Layer)

FC layer, often referred to as a dense layer, is prevalent in deep learning models. Each neuron or node in this layer is attached to all neurons in the previous layer, forming a fully connected structure. The fully connected layer can receive and integrate all features from the previous layer. These features, after being processed by earlier layers, carry important information about the input data. The fully connected layer further abstracts and integrates this information through linear weighted combinations and nonlinear activation functions, preparing it for the final classification or regression tasks.

#### 2.2.5. Attention Mechanism

The attention mechanism is a capability of the nervous system to screen and focus on information. It allows organisms to quickly identify important information in complex environments while ignoring irrelevant information. The self-attention mechanism in deep learning draws inspiration from some characteristics of how organisms process information, including dynamism, selectivity, and integration, which collectively constitute the core of the self-attention mechanism. The attention mechanism has a broad spectrum of applications in multiple fields, such as computer vision, and sequence modeling. The attention mechanism allows each element in the input sequence to be compared with other elements in the sequence to compute a representation of the sequence. This mechanism enables the model to focus on the relationships between different positions in the input sequence, thereby capturing complex dependencies within the sequence.

The fundamental concept of the dot-product attention mechanism is to assign attention weights based on the similarity between queries and key-value pairs. The dot-product attention mechanism is capable of capturing the correlations between different parts of the input sequence, which is crucial for understanding the context of the input data, capturing important features, and improving the performance of the model. The proposed model adopts a dual-channel structure, where one channel is a convolutional neural network (CNN) used for extracting spatial features, and the other channel is a bidirectional long short-term memory network (BiLSTM) used for extracting temporal sequence features. The outputs from both channels are combined through concatenation to form a merged feature vector, which is then passed as input to the attention mechanism layer. In the dot-product attention mechanism, the input features are first mapped into three spaces: query (Q), key (K), and value (V). These mappings are achieved by applying fully connected layers to the input features. The query (Q) is obtained through a linear transformation for comparison with the key (K). The key (K) is also derived from a linear transformation for comparison with the query (Q) to compute similarity. The value (V) represents the original input features and is typically passed directly to participate in the final weighted summation.

First, calculate the similarity between the query (Q) and the key (K). The specific calculation process is as follows in Equation (9):

(9) $$\mathrm{Attention}\;\mathrm{Score}=\frac{Q\cdot K^{T}}{\sqrt{d_{k}}}$$

In Equation (9), Q is the query vector, *K^T^* is the transpose of the key vector, and *d_k_* is the dimensionality of the key vector. This step computes the similarity score between each pair of queries and keys.

Then, the obtained attention scores are processed through the *Softmax* function to convert them into a probability distribution, resulting in normalized attention weights, as shown in Equation (10):

(10) $$\mathrm{Attention}\;\mathrm{Weight}=\mathrm{Softmax}(\frac{Q\cdot K^{T}}{\sqrt{d_{k}}})$$

Finally, the computed attention weights are used to perform a weighted sum of the values (V), resulting in the final output, as shown in Equation (11):

(11) Output=Attention Weight⋅V

This process ensures that the model focuses on those features that are more critical to the task while suppressing attention to irrelevant features. An attention mechanism is introduced into the proposed model, allowing the weighted fusion of features to depend not only on the spatial and temporal information of the input features but also to dynamically select the most meaningful features according to the requirements of the task.

### 2.3. Evaluation Indexes

This study uses EEG signals for emotion classification and adopts five key metrics to assess model performance. They are accuracy, recall, precision, F1-score, and Matthews correlation coefficient (MCC), which comprehensively analyze the model’s effectiveness and stability. The calculating Equations (12)–(16) are as follows. Among them, true positives (TPs) refer to the number of samples correctly predicted as positive by the model. False positives (FPs) indicate the number of samples incorrectly predicted as positive by the model. True negatives (TNs) represent the number of samples correctly predicted as negative by the model. False negatives (FNs) refer to the number of samples incorrectly predicted as negative by the model. Accuracy indicates the proportion of samples that the model predicted correctly out of the total samples. Precision represents the proportion of actual positive samples among all samples predicted as positive by the model. Recall indicates the proportion of correctly predicted positive samples among all actual positive samples. The F1-score is the harmonic mean of precision and recall, used to comprehensively consider the balance between recall and precision. The MCC provides a comprehensive measure of model performance by considering true negatives, false positives, true positives, and false negatives.

(12) $$accuracy=\frac{TP+TN}{TP+FP+TN+FN}$$

(13) $$precision=\frac{TP}{TP+FP}$$

(14) $$recall=\frac{TP}{TP+FN}$$

(15) $$F1-score=\frac{2}{\frac{1}{precision}+\frac{1}{recall}}$$

(16) $$MCC=\frac{TP\times TN-FP\times FN}{\sqrt{\left(TP+FP)(TP+FN)(TN+FP)(TN+FN\right)}}$$

## 3. Experimental Results and Analysis

### 3.1. Experimental Setup

When processing EEG data, we observed a notable temporal correlation within the data, indicating that EEG signals from the same individual may exhibit strong dependencies across different time points. If using data from the same individual as both the training set and the test set, this could lead to information leakage and bias the results of model evaluation. Therefore, in a single test, the SEED and SEED-IV dataset are divided into approximately 80% of the data (data from twelve individuals) as the training set, and the remaining approximately 20% of the data (data from three individuals) as the test set, ensuring that the training set and the test set come from different individuals, which effectively prevents the issue of data leakage. In this way, model evaluation results can be obtained based on a single data partition. However, considering the randomness of data partitioning, the results of a single test may not comprehensively reflect the model’s generalization ability. To further validate the robustness of the model, a 10-fold cross-validation experiment is also conducted. In the 10-fold cross-validation, the dataset is divided into ten subsets (folds), using nine subsets for training each time and the remaining one subset as the validation set. This process is repeated 10 times, each time selecting a different validation set. In the end, the results of the 10 experiments are averaged as a measure of the model’s performance. Cross-validation not only helps avoid biases that may arise from a single data split but also effectively enhances the model’s generalization ability, ensuring its stability across different data partitions. Table 1 shows the parameter settings of the MSBiLSTM-Attention model used in this study. In this paper, a random search method is used to select the optimal configuration by traversing different combinations of hyperparameters. Specifically, we evaluate each hyperparameter combination with K-fold cross-validation to ensure that the selected hyperparameter configuration performs well on the validation set.

### 3.2. Single Test Result of MSBiLSTM-Attention Model

This study aims to evaluate the performance of the proposed MSBiLSTM-Attention model in recognizing emotion signals in EEG data and to demonstrate its effectiveness by comparing it with several existing deep learning and traditional machine learning algorithms. The single test performance of the MSBiLSTM-Attention model will be comprehensively assessed. Specifically, a convolutional neural network-gated recurrent unit (CNN-GRU), a convolutional neural network-long short-term memory network (CNN-LSTM), a one-dimensional convolutional autoencoder (1D CAE), 1D Inception, EEGNet [40], and VGG16-LSTM [41] are selected as representatives of deep learning algorithms for comparative experiments. In addition to deep learning methods, this study also considers traditional machine learning algorithms, including Adaboost and Bayes.

A single test of the MSBiLSTM-Attention model was employed to perform three-classification on the SEED dataset and four-classification on the SEED-IV dataset, respectively, and was relative to other models, with the outcomes presented in Table 2 and Table 3. On the SEED dataset, the MSBiLSTM-Attention achieved an accuracy of 99.44%, precision of 99.44%, recall of 99.44%, F1-score of 99.43%, and MCC of 99.16%. The VGG16-LSTM, coming in just after the MSBiLSTM-Attention, had an accuracy of 98.01%, precision of 98.02%, Recall of 98.01%, F1-score of 98.01%, and MCC of 97.02%. The EEGNet method had the worst results, with an accuracy of 37.56%, precision of 36.65%, recall of 37.56%, F1-score of 36.17%, and MCC of 6.5%. On the SEED-IV dataset, the accuracy of MSBiLSTM-Attention is 99.85%, precision is 99.85%, recall is 99.85%, F1-score is 99.85%, and MCC is 99.80%. The accuracy of VGG16-LSTM is 96.79%, precision is 96.83%, recall is 96.79%, F1-score is 96.79%, and MCC is 95.21%.

### 3.3. 10-Fold Cross-Validation Results of MSBiLSTM-Attention Model

The average result of 10-fold cross-validation of the MSBiLSTM-Attention model was employed to perform three-classification on the SEED dataset and four-classification on the SEED-IV dataset, respectively, and was relative to other models, with the outcomes summarized in Table 4 and Table 5. As shown in Table 4, the proposed MSBiLSTM-Attention model demonstrates remarkable performance, with an accuracy of 99.49%, precision of 99.50%, recall of 99.49%, F1-score of 99.49%, and MCC of 99.24%. As shown in Table 5, the MSBiLSTM-Attention model achieved an accuracy of 99.70%, precision of 99.70%, recall of 99.70%, F1-score of 99.70%, and MCC of 99.69%.

Therefore, based on the results from Table 1, Table 2, Table 3 and Table 4, it can be concluded that compared to other comparative models, the MSBiLSTM-Attention model demonstrates excellent performance in the task of emotion recognition classification using EEG signals, both in single validation and 10-fold cross-validation. This verifies the stability and reliability of the MSBiLSTM-Attention model.

### 3.4. Ablation Experiment

To explore the value of synchronously extracting spatiotemporal information in multi-channel models, this study performed ablation experiments on the SEED and SEED-IV datasets. Specifically, Block1 excluded the Bi-LSTM temporal feature extraction component of Block2, while Block2 removed the multi-scale convolutional spatial feature extraction module of Block1, ensuring that the remaining architecture components remained unchanged. The results of the ablation tests, detailed in Table 6 and Table 7, clearly show that the performance of the single-channel spatial feature module is inferior to that of our MSBiLSTM-Attention model. From Table 6 and Table 7, it is evident that the Block2 component of the single-channel setup performs better than Block1, which may be due to the greater importance of temporal features over spatial features in EEG data. This analysis not only emphasizes the importance of synchronously processing spatiotemporal information for emotion recognition tasks but also highlights the robust capability of the MSBiLSTM-Attention model in handling complex scenarios, providing strong evidence for future research.

## 4. Conclusions

This study presents a new MSBiLSTM-Attention model aimed at addressing the challenges of emotion classification based on EEG signals. Specifically, it combines the spatial feature extraction capability of multi-scale convolution, the temporal sequence processing advantages of Bi-LSTM, and the efficient information-filtering function of the attention mechanism. Experimental results show that the model achieves an accuracy of 96.67% on the SEED dataset and 99.70% on the SEED-IV dataset. The MSBiLSTM-Attention model not only meticulously analyzes the spatiotemporal characteristics of EEG signals but also intelligently selects the electrode channels that have the most significant impact on prediction results, highlighting its potential for automated EEG-based emotion recognition. The transformer model is not used in this paper because of the lack of induction bias compared to CNN [42], so a large amount of data is required for training. The transformer structure is bulky, with many parameters and flop, which is not conducive to practical application. In the future, it is also planned to further optimize the computational efficiency of the model in our work. For example, the use of more efficient network architectures (such as depthwise separable convolutions) or exploring new training techniques (such as adaptive learning rate adjustments and hardware optimizations) is being considered to further reduce training time. Additionally, we can perform multimodal fusion of MSBiLSTM-Attention with other signals such as electromyography and eye movement, which can further enhance the model’s accuracy. Finally, there are significant differences in the subject data of the SEED dataset, leading to fluctuations in the experimental accuracy between different subjects. Therefore, reducing the feature distribution disparity between subjects is also a topic worth exploring [43].

## Figures and Tables

**Table 1:** Parameter settings for the MSBiLSTM-Attention model.

Parameter	Value
epoch number	100
learning rate	0.001
batch size	1024
optimizer	Adam
loss function	categorical_cross-entropy
convolution kernel	32
activation function	ReLU
multi-scale convolution	1 × 1, 1 × 3
Bi-LSTM	32
FC1	64
FC2	32
classifier	Softmax
random seed	42

**Table 2:** The performance of MSBiLSTM-Attention model based on single validation in three classification tasks.

Methods	Accuracy (%)	Precision (%)	Recall (%)	F1-Score (%)	MCC (%)
CNN-GRU	70.38	70.42	70.38	70.32	55.64
CNN-LSTM	94.65	94.67	94.65	94.65	92.00
1D CAE	96.01	96.06	96.01	96.01	94.05
1D InceptionV1	86.65	86.71	86.65	86.64	80.01
EEGNet	37.56	36.65	37.56	36.17	6.5
VGG16-LSTM	98.01	98.02	98.01	98.01	97.02
Adaboost	54.29	55.03	54.29	53.99	31.86
Bayes	40.95	42.97	40.95	35.88	13.77
MSBiLSTM- Attention	99.44	99.44	99.44	99.43	99.16

**Table 3:** The performance of MSBiLSTM-Attention model based on single validation in four classification tasks.

Methods	Accuracy (%)	Precision (%)	Recall (%)	F1-Score (%)	MCC (%)
CNN-GRU	56.94	57.54	56.94	56.70	42.85
CNN-LSTM	84.90	84.97	84.90	84.89	79.90
1D CAE	88.45	88.54	88.45	88.46	84.62
1D InceptionV1	77.96	78.14	77.96	77.94	70.68
EEGNet	26.45	26.59	26.45	25.29	2.1
VGG16-LSTM	96.79	96.83	96.79	96.79	95.21
Adaboost	37.49	37.52	37.49	37.41	16.69
Bayes	26.10	30.44	26.10	17.39	2.46
MSBiLSTM- Attention	99.85	99.85	99.85	99.85	99.80

**Table 4:** The performance of MSBiLSTM-Attention model based on ten-fold cross-validation in three classification tasks.

Methods	Accuracy (%)	Precision (%)	Recall (%)	F1-Score (%)	MCC (%)
CNN-GRU	81.67	81.82	81.67	81.67	72.57
CNN-LSTM	94.86	94.87	94.86	94.85	92.30
1D CAE	93.65	93.67	93.65	93.65	90.49
1D InceptionV1	88.32	88.37	88.32	88.31	82.51
EEGNet	45.46	46.34	45.47	44.91	18.62
VGG16-LSTM	93.98	94.01	93.98	93.99	90.98
Adaboost	52.63	53.38	52.64	52.35	29.35
Bayes	41.79	42.23	41.79	38.82	13.75
MSBiLSTM- Attention	99.49	99.50	99.49	99.49	99.24

**Table 5:** The performance of MSBiLSTM-Attention model based on ten-fold cross-validation in four classification tasks.

Methods	Accuracy (%)	Precision (%)	Recall (%)	F1-Score (%)	MCC (%)
CNN-GRU	58.55	58.87	58.55	58.43	44.88
CNN-LSTM	84.13	84.18	84.13	84.12	78.86
1D CAE	84.13	84.21	84.13	84.13	78.87
1D InceptionV1	76.55	76.68	76.55	76.54	68.78
EEGNet	41.94	46.79	41.93	40.67	23.75
VGG16-LSTM	98.28	98.28	98.28	98.28	97.71
Adaboost	35.93	35.98	35.93	35.82	14.61
Bayes	25.77	28.84	25.77	17.34	1.66
MSBiLSTM- Attention	99.70	99.70	99.70	99.70	99.60

**Table 6:** Ablation experiments of MSBiLSTM-Attention model based on ten-fold cross-validation (SEED dataset).

Methods	Accuracy (%)	Precision (%)	Recall (%)	F1-Score (%)	MCC (%)
Block1	98.24	98.25	98.24	98.24	97.37
Block2	99.04	99.04	99.04	99.04	98.56
MSBiLSTM- Attention	99.49	99.50	99.49	99.49	99.24

**Table 7:** Ablation experiments of MSBiLSTM-Attention model based on ten-fold cross-validation (SEED-IV dataset).

Methods	Accuracy (%)	Precision (%)	Recall (%)	F1-Score (%)	MCC (%)
Block1	97.50	97.50	97.49	97.49	96.67
Block2	98.22	98.22	98.22	98.22	97.63
MSBiLSTM- Attention	99.70	99.70	99.70	99.70	99.60

## Data Availability

Restrictions apply to the availability of these data. Data were obtained from [BCMI laboratory] and are available from the authors/at https://bcmi.sjtu.edu.cn/~seed/index.html (accessed on 20 February 2025).
